# Mandibular Overdentures on Four Immediately Loaded Mini‐Implants Versus Two‐Delayed Loaded Regular Implants: 1‐Year, Two‐Center RCT on Patient‐Reported Outcomes

**DOI:** 10.1111/clr.70143

**Published:** 2026-06-01

**Authors:** Martin Schimmel, Cláudio Rodrigues Leles, Nicole Schenk, Simone F. M. Janner, Beat Wallkamm, Valérie G. A. Suter, Samir Abou‐Ayash, Clemens Raabe

**Affiliations:** ^1^ Department of Reconstructive Dentistry and Gerodontology, School of Dental Medicine University of Bern Bern Switzerland; ^2^ School of Dentistry Federal University of Goias Goiania Goias Brazil; ^3^ Surgery Center ZIKO Bern Bern Switzerland; ^4^ Department of Oral Surgery and Stomatology School of Dental Medicine, University of Bern Bern Switzerland; ^5^ Division of Periodontology School of Dental Medicine, University of Geneva Geneva Switzerland; ^6^ Private Practice Langenthal Switzerland; ^7^ Department of Prosthetic Dentistry and Material Science University Medical Center of the Johannes Gutenberg University Mainz Mainz Germany; ^8^ Department of Oral Surgery and Implantology Goethe University, Carolinum Frankfurt am Main Germany

## Abstract

**Objective:**

This one‐year randomized clinical trial compared the effects of mandibular implant‐overdentures (IODs) on two two‐piece regular‐neck tissue‐level implants (Group A) or on four mini‐implants (Group B) with different loading protocols in edentulous patients concerning dental Patient‐Reported Outcomes (dPROs).

**Material and Methods:**

Seventy‐two participants were randomly assigned to Group A (*n* = 35) for treatment with two standard implants (3.3 mm diameter, conventional loading) with a stud‐type abutment, or to Group B (*n* = 37) for treatment with four mini‐implants (2.4 mm diameter, immediate loading) with a mini‐stud‐type abutment. Primary outcomes were measured using the General Oral Health Assessment Index (GOHAI), the 14‐item Oral Health Impact Profile (OHIP‐G14), and the Denture Satisfaction Index (DSI), at visit #1—baseline (pre‐treatment), visit #2—implant placement (immediate loading, Group B), visit #3–8‐10‐week follow‐up (conventional loading, Group A; control visit, Group B), and visit #4–1‐year post‐placement. Data analysis included descriptive statistics, non‐parametric comparison tests, effect‐size measures, and multiple regression.

**Results:**

Sixty‐two participants completed the 1‐year follow‐up (Group A = 32; Group B = 30). Significantly improved dPROs were observed for both treatments after 1 year, compared with baseline (*p* < 0.01), with similar effects in the two Groups. In the mini‐implant group, higher improvement in satisfaction was observed after implant placement and immediate loading, compared to Group A (*p* < 0.001).

**Conclusion:**

Significant improvements in satisfaction and Oral Health Related Quality of Life were observed after 1 year, and benefits were observed earlier for mini‐implants using immediate loading. Overall, findings support the use of four immediately loaded mini‐implants in the mandible as a suitable treatment alternative for edentulous patients.

**Registration:**

ClinicalTrials.Gov ID: NCT03837158. https://clinicaltrials.gov/study/NCT03837158.

## 1 Introduction

The use of two implants for a mandibular implant‐overdenture (IOD) opposing a complete maxillary denture has been regarded as the minimum standard of implant treatment for the edentulous patient (Feine et al. 2002; Thomason et al. 2009; Schimmel et al. 2023). This approach provides improved patient benefits compared to conventional complete dentures as a result of superior stability and retention and improved oral function (Klemetti et al. 2003; Walton 2003; Abou‐Ayash et al. 2023). Nevertheless, several alternatives varying the number and type of implant systems have been proposed to optimize treatment procedures, minimize treatment burdens, and improve patient experience (Stoker et al. 2007; Meijer et al. 2009; Lee et al. 2012; Raabe et al. 2024).

An alternative approach using narrow‐diameter implants, namely “mini‐implants” with < 2.5 mm diameter, has been used to reduce the morbidity of the surgical intervention, providing less invasive and straightforward interventions, especially for elder patients and those with limited alveolar width (Klein et al. 2014). Previous studies have reported satisfactory clinical and dental Patient‐Reported Outcomes (dPROs) with mini‐implant retained IODs (Reissmann et al. 2018; Enkling et al. 2019), although implant survival has been considered lower compared to conventional implants (Schiegnitz and Al‐Nawas 2018).

Studies with early mini‐implant systems revealed important biomechanical and biological limitations, including increased implant fracture, greater marginal bone loss, compromised long‐term stability, and increased prosthetic maintenance, raising concerns about their indiscriminate use (Shatkin et al. 2007; Bidra and Almas 2013). Mini‐implants showed inferior outcomes compared with conventional implants, mostly due to overloading, insufficient implant numbers, and inappropriate case selection, reinforcing that early mini‐implant failures were largely technique‐ and indication‐dependent (Bidra and Almas 2013; De Souza et al. 2015; Schiegnitz and Al‐Nawas 2018).

Aiming to overcome the limitations of early mini‐implant systems, in 2018, a novel titanium‐zirconium mini‐implant system was launched with improved characteristics (Straumann Mini Implant System, Institut Straumann AG, Basel, Switzerland). It is a 2.4 mm one‐piece, apically tapered implant body design with a tissue‐level neck, made of a high‐strength titanium‐zirconium alloy (Roxolid) and a sandblasted, large grit, acid‐etched implant surface (SLA), and a prosthetic connection coated with an amorphous diamond‐like carbon (ADLC) surface that attaches to a PEEK (polyetheretherketone) matrix insert in a titanium housing (Straumann Optiloc Retentive System, Institut Straumann AG, Basel, Switzerland) (Curado et al. 2023). It is a miniaturized version of the Straumann Novaloc Retentive System Institut Straumann AG, (Basel, Switzerland). These characteristics aim to improve implant stability and overall survival, as well as peri‐implant health and effective, long‐term prosthetic retention, and allow for patient‐centered treatment protocols, such as immediate implant loading. A previous randomized clinical trial reported high implant survival and peri‐implant bone stability (Curado et al. 2023), satisfactory patient satisfaction (Curado et al. 2024) and prosthetic outcomes (Silva et al. 2024), even when flapless surgery and immediate loading were adopted (Leles et al. 2022).

This randomized clinical two‐center trial aimed to assess the comparative effectiveness of treatments with mandibular overdentures retained by four mini‐implants and the standard treatment using two implants. Treatment effectiveness was evaluated based on changes in dPROs before and after treatment. The study's main hypothesis is that within the first year post‐placement, four interforaminally placed, immediately loaded 2.4 mm one‐piece mini‐implants with a mini‐stud‐type (Optiloc) attachment show improved Patient Reported Outcome Measures (PROMs) compared to two interforaminally placed, conventional loaded, 3.3 mm two‐piece regular‐neck implants with regular stud‐type (Novaloc) attachments to retain a mandibular IOD. The ultimate goal of the study was to compare the two treatment alternatives to evaluate which one is better recommended for edentulous patients.

## 2 Material and Methods

### 2.1 Study Design and Interventions

This is a 1‐year, two‐group, parallel randomized two‐center clinical trial, comparing the treatment with a mandibular overdenture retained by two TiZr two‐piece regular‐diameter implants with stud‐type attachments (control group—Group A) and four one‐piece TiZr mini‐implants with stud‐type attachments (experimental group—Group B). The retentive parts in Group A have a connection with separate screwed standard‐sized Novaloc abutment and a wear‐resistant Amorphous Diamond‐Like Carbon (ADLC) coating, connected to a female part composed of a metal denture cap and interchangeable PEEK retention inserts. The one‐piece mini‐implants in Group B are fitted with a size‐reduced version of the Novaloc retentive attachment (Optiloc). The two systems are identical in all features except for their dimensions. The study adopted a 1:1 allocation ratio and a superiority trial framework.

This report followed the CONSORT 2025 guidelines for randomized clinical trials (Hopewell et al. 2025; Data S1). The original study protocol was registered at the ClinicalTrials.gov database on 06/02/2019 under the identification NCT03837158 (https://clinicaltrials.gov/study/NCT03837158). The study was also approved by the Swiss Ethics Committees on research involving humans (Swissethics ID 2019–00408), in accordance with the current version of the World Medical Association Declaration of Helsinki, ICH‐GCP guidelines, ISO 14155, and the local legally applicable requirements. The study is a two‐center trial conducted at the clinical settings School of Dental Medicine, University of Bern, Switzerland, and a private dental practice (Dr. Beat Wallkamm, Langenthal, Switzerland). This study received external funding from the International Team for Implantology (ITI grant 1286_2018). Surgeries for implant placement were conducted between May 2020 and August 2023.

### 2.2 Study Sample, Enrollment, and Randomization

Eligible participants were edentulous adults rehabilitated with conventional complete dentures over a period of at least 6 months. Participants were recruited from the Department of Reconstructive Dentistry and Gerodontology, the Department of Oral Surgery and Stomatology, School of Dental Medicine at the University of Bern, and a private practice in the vicinity of Bern (Dr. med. dent. Beat Wallkamm, Langenthal, Canton of Bern, Switzerland). To be included in the study, participants had to meet the inclusion and exclusion criteria, as follows:

Inclusion Criteria:

- At the patient level: age ≥ 18 years, objectively satisfactory complete dentures (Heyink et al. 1986) that are not older than 1 year, subjective dissatisfaction with the mandibular denture to the degree of seeking treatment; ASA physical status PS1 and PS2 (American Society of Anesthesiologists).
- At implant site level: healed edentulous mandible (minimum 6 months since last extraction), minimal ridge dimensions 5.5 mm (width) by 12 mm (height) in the anterior (interforaminal) area, allowing the placement of at least 3.3 mm (endosseous diameter) by 10 mm (length) regular‐neck tissue‐level implants, opposing dentition consisting of a complete denture on an edentulous maxilla.

Exclusion criteria:

- At patient level: any physical or mental disorder that would interfere with the ability to perform adequate oral hygiene or the capability of providing written informed consent and compliance to the protocol, any disorder that would interfere with wound healing or represent a contraindication for oral surgery such as, but not limited to, uncontrolled diabetes or conditions resulting in or requiring immunosuppression, radiation, chemotherapy, frequent use of antibiotics or antiresorptive medication such as bisphosphonates; pregnancy or lactation; heavy smoking habit with > 20 cigarettes per day; severe bruxism or clenching habits, present orofacial pain.
- At site level: ridge defects requiring horizontal and/or vertical bone augmentation procedures.

The participants were evaluated for initial study eligibility during the screening visit (visit #1). Those who were eligible according to the inclusion and exclusion criteria and were willing to participate were asked to sign an informed consent form and were subsequently enrolled in the study. All patients had to be willing to return to the study centers to complete these visits within 1 year after overdenture treatment delivery. Patients paid a flat fee towards the treatment.

After enrollment, participants were randomized into the two treatment Groups using block randomization of eight patients, by means of an online randomization software (randomizer.org). The assignment to interventions was performed by an independent research nurse (BG) not involved in patient care. The differences between the Groups included implant‐diameter (2.4 mm (Group B) vs. 3.3 mm (Group A)), number of implants (4 (Group B) vs. 2 (Group A)), implant‐design (one‐piece (Group B) vs. two‐piece (Group A)), and implant loading protocol (immediate (Group B) vs. conventional (Group A)), with implant material and surface being identical.

A priori sample size calculation was based on previous reports on PROMS in the literature (Fillion et al. 2013; Batisse et al. 2017). Accordingly, in each Group, 36 participants would be enrolled to reject the null hypothesis of difference between treatments. Then, a post hoc power analysis (G*Power 3.1.9.4) for a repeated‐measures design (within‐between interaction) revealed that this sample size was sufficient to detect a small effect size (0.15) with 0.80 power and 0.05 alpha error.

### 2.3 Interventions

Surgical diagnosis was based on a preoperative examination, during which a thorough evaluation of the patient's medical and dental history was conducted, along with clinical and radiological assessments. Radiological analysis of the anterior mandible was performed using a Cone‐Beam Computed Tomography (CBCT) scan with an 8 × 5 cm field of view, voxel size of 160 μm and exposure setting of 5.0 mA and 90 kV (3D Accuitomo 170, J. Morita Corp, Osaka, Japan). The CBCTs were evaluated within the software program i‐Dixel.

The implant surgery (visit #2) was performed under local anesthesia with respect to critical anatomic structures, such as the mental foramen or lingual undercuts. Surgery was initiated by a mid‐crestal incision and elevation of a minimal mucoperiosteal flap, and vertical reduction/osteotomy of the alveolar crest was then carried out if indicated. Implant placement was conducted following the manufacturer's recommendations, with the existing denture used for intraoperative orientation, alignment of the implant osteotomies and prosthodontically correct 3D implant positioning. Patients in Group A received two 3.3 mm tissue‐level implants each (Standard Implants, diameter 3.3 mm, Institut Straumann AG, Switzerland) (RN), whilst patients in Group B received a total of four 2.4 mm tissue‐level mini‐implants each (Straumann Mini Implant System, Institut Straumann AG, Switzerland) (Raabe et al. 2025). Implant insertion torque was measured during implant placement as an indicator for primary implant stability. For both Groups, flap closure using single‐interrupted sutures and transmucosal healing was carried out, including appropriate healing abutment selection for two‐piece implants (Group A).

In Group A, the existing denture was adapted to facilitate correct wound healing and prevent premature implant loading.

In Group B, an insertion torque of ≥ 25 Ncm was defined as sufficient primary stability to allow for immediate loading (Schimmel et al. 2014). The retentive parts (Straumann Optiloc Retentive System, Institut Straumann AG, Basel, Switzerland) were incorporated in the pre‐existing prosthesis with self‐curing PMMA resin (Unifast TRAD, GC Corporation, Lucerne, Switzerland) intraorally using a chairside procedure (Leles et al. 2022). If any of the mini‐implants did not achieve the minimum primary stability for immediate loading (≥ 25 Ncm insertion torque), they were left unloaded for 10–12 weeks.

Post‐surgical visits were scheduled 7–15 days after implant placement (1–2 visits for suture removal and denture adaptation). During healing, the denture was relined with a soft silicone (GC ReLine Soft, GC Corporation, Lucerne, Switzerland). These patients were reported in the Case Report Form (CRF) and analyzed according to the Intention‐To‐Treat (ITT) principle.

After 8–10 weeks, patients from Group A were invited to return (visit #3). Implants were conventionally loaded by placing stud‐type abutments (Straumann Novaloc Retentive System, Institut Straumann AG, Basel, Switzerland), with the appropriate height. The housings were incorporated in the pre‐existing prosthesis indirectly after taking a reline impression with Identium (Kettenbach GmbH, Eschenburg, Germany). A closed mouth reline impression procedure in maximum intercuspation was followed. The converted prosthesis was delivered the same day; within 6 h for Group B, a follow‐up examination was scheduled, or in case of insertion torques < 25 Ncm, implant loading was carried out as described.

**FIGURE 1:**
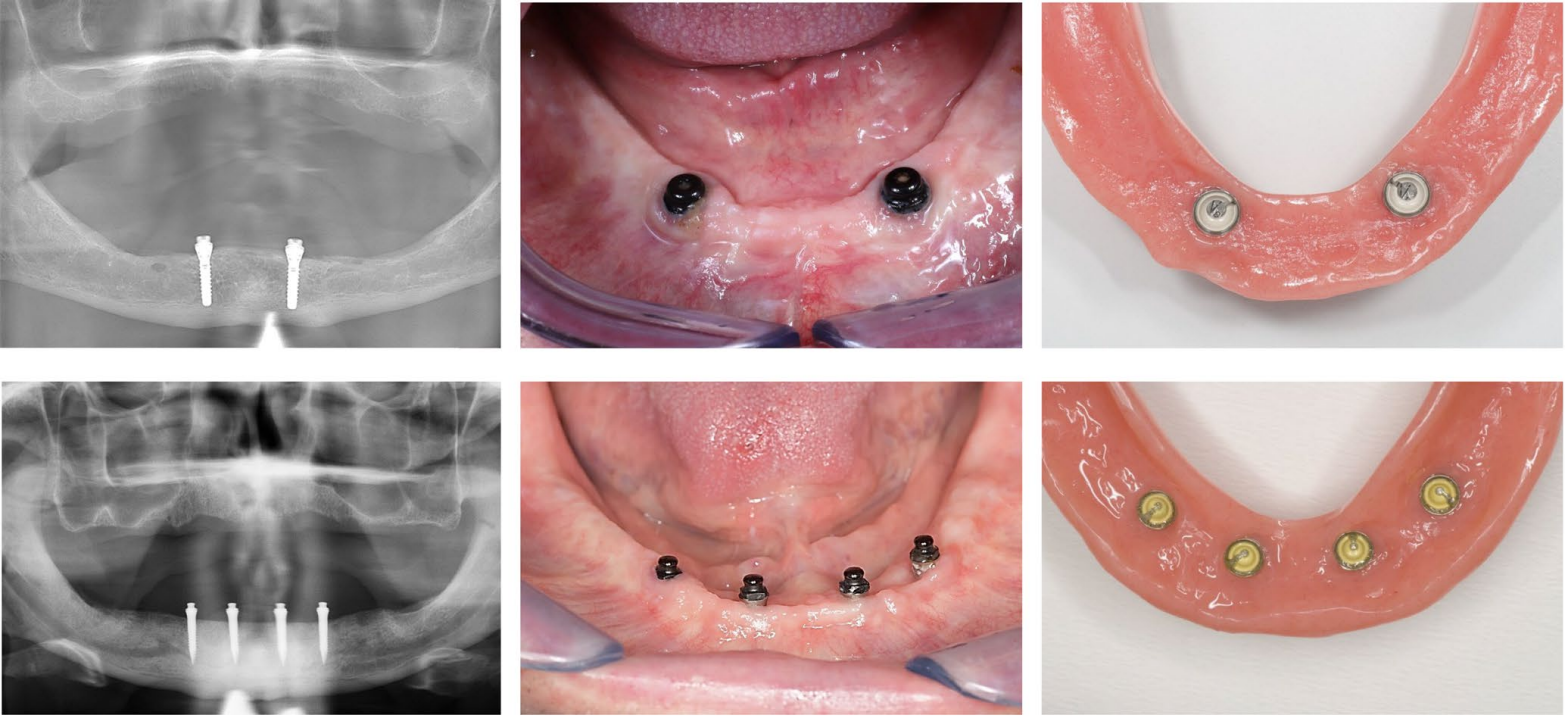
Clinical and radiographic aspects of the two‐implant mandibular overdenture—Group A (upper), and the four mini‐implant mandibular overdenture—Group B (lower).

In the two Groups, 1 year after implant loading, participants were invited to return for the one‐year follow‐up visit (visit #4). A clinical presentation of the mini‐implant treatment at the one‐year follow‐up is shown in Figure 1.

At the time of implant loading, all matrix housings in both groups were fitted with the white PEEK retention insert (light retention, approximately 750 g), as recommended by the manufacturer. The colour‐coded retention insert system is identical for the Novaloc (Group A) and Optiloc (Group B) attachments, allowing a direct comparison of retention force between groups. Inserts were exchanged for a stronger version (white → yellow → green → blue, corresponding to approximately 750 g, 1200 g, 1650 g, and 2100 g of retention) only if a participant reported insufficient denture retention at a recall visit; the retention force was never reduced. At the one‐year follow‐up, the majority of participants in both groups had been transitioned from white to yellow inserts, while a minority required green or blue inserts. The frequency of insert exchanges did not differ meaningfully between groups.

### 2.4 Outcomes

The primary treatment effects were assessed by PROMs: the General Oral Health Assessment Index—GOHAI (Hassel et al. 2008), the 14‐item version of the Oral Health Impact Profile—OHIP‐G14 (John et al. 2002), and the Denture Satisfaction Index—DSI (Awad and Feine 1998). Data collection of outcome measures was planned to assess post‐treatment changes from baseline (pre‐treatment).

The German validated translation of the GOHAI was used to assess Oral Health‐related Quality of Life (OHRQoL) (Hassel et al. 2008), as an index of the impact of oral disorders on health‐related quality of life, by calculating an overall score to indicate the extent of a range of functional and psycho‐social consequences. GOHAI contains 12 statements (e.g., “How often did you feel uncomfortable eating in front of people because of problems with your teeth or dentures”) with a Likert response format (i.e., 0 = never, 1 = seldom, 2 = sometimes, 3 = often, 4 = very often, 5 = always). Response codes were summed across the 12 statements to yield an overall score ranging from 0 to 60 (Allen 2003). Higher values mean better OHRQoL.

The OHIP‐G14 instrument covers the four domains of orofacial pain, oral functions, psychosocial impact, and appearance, and is suitable for the application in cross‐sectional as well as longitudinal studies to assess OHRQoL (John et al. 2006; Allen and Locker 2002). It is based on the OHIP‐49 (Slade and Spencer 1994) and includes items relating to perceived chewing difficulty. For each question, participants were asked how frequently they had experienced the event during the last month. Responses are given on a 5‐point Likert scale, where lower values mean better OHRQoL.

Denture satisfaction was evaluated using a 10‐mm visual analogue scale (VAS)‐based questionnaire, namely the Denture Satisfaction Index (DSI) (Awad and Feine 1998). DSI evaluates nine different items that cover the ease of cleaning, general satisfaction, speech, comfort, aesthetics, stability, chewing ability, function, and the general oral condition. Some items have sub‐items, resulting in a total of 23 10 mm‐VAS scales.

### 2.5 Data Analysis

Data was summarized using descriptive statistics. Normality of the data was tested using the Shapiro–Wilk test, and bivariate comparisons were used to assess changes from baseline across the different time points (within‐patient) and to compare the two study Groups (between‐patients) at each time point. Additionally, the magnitude of changes was assessed by calculating the treatment effect sizes for each Group at the 1‐year follow‐up compared to baseline. For the non‐parametric Wilcoxon signed‐rank test, “*r*” is a standardized effect size statistic calculated by dividing the z‐statistic by the square root of the number of pairs (z/sqrt(N)). The reference parameters for interpretation of the effect sizes were: |*r*| < 0.1 is no effect or very small effect, 0.1 ≤ |*r*| < 0.3 is a small effect, 0.3 ≤ |*r*| < 0.5 is a moderate effect, and |*r*| ≥ 0.5 is a large effect.

Multiple regression analysis using Generalized Estimating Equations (GEE) was employed to evaluate the impact of time and study Groups on treatment outcomes. GEE was performed due to the repeated measurements of the longitudinal data, and to handle missing data, assuming that missing values occurred completely at random and unrelated to the observed data. The effect of time points was tested, using the baseline as a reference, and controlling for the two study settings. A normal distribution and an identity link function were used, since GEE is a semi‐parametric approach that allows for non‐normal or skewed outcomes by focusing on the mean rather than the full probability distribution of the data, making it robust to distributional assumptions about the outcome. The selected working correlation structure was Autoregressive (AR‐1), which assumes correlation decreases over time, commonly used for longitudinal data, assuming that measurements closer in time are more strongly correlated than those further apart (correlation decreases exponentially as the time distance between measurements increases). The goodness‐of‐fit of the model was assessed by comparing competing GEE models with different parameter settings (working correlation structures, presence vs. absence of interactions, and different predictor sets) fitted to the same data. Regression model estimates were expressed as regression coefficients and their 95% confidence intervals. The IBM SPSS 24.0 software was used for statistical analysis, and the level of significance for statistical inference was set at *p* < 0.05.

## 3 Results

**FIGURE 2:**
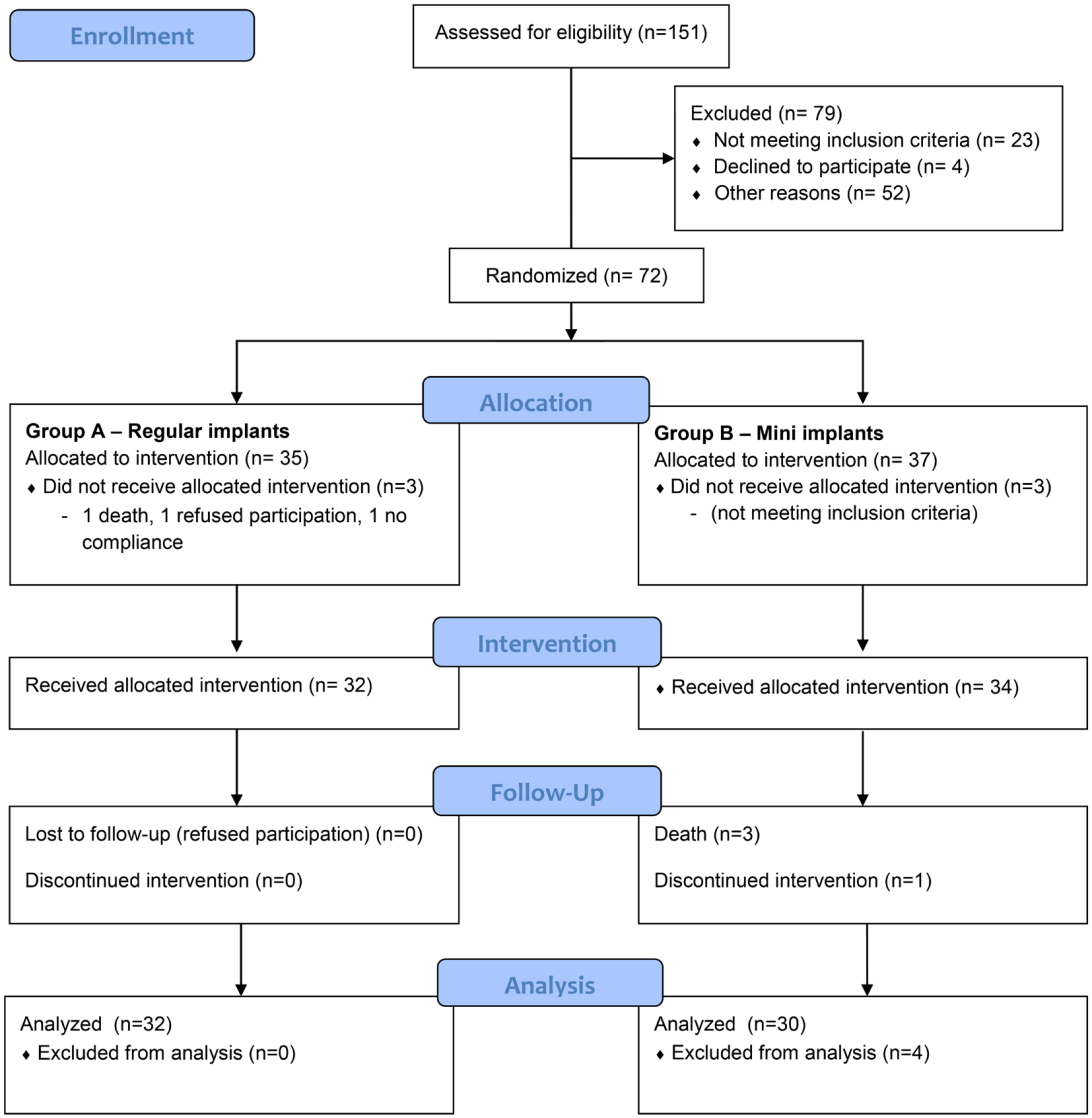
Patient flow diagram.

From a total of 151 eligible participants screened for eligibility, 72 were selected and randomized to the study Groups, comprising 43 (59.7%) males and 29 (40.3%) females, with ages ranging from 40 to 94 years (mean = 68.0; SD = 11.3). The Group assignment resulted in 35 participants in Group A and 37 in Group B. The two Groups were similar with respect to sex (*p* = 0.058) and age (*p* = 0.562). In Group A, 32 participants and in Group B, 34 participants received the assigned intervention. All participants from Group A completed the 1‐year follow‐up. In Group B, four participants were excluded due to death (*n* = 3) or study withdrawal (*n* = 1), and 30 completed the 1‐year follow‐up. The complete patient flow diagram throughout the study is shown in Figure 2.

**FIGURE 3:**
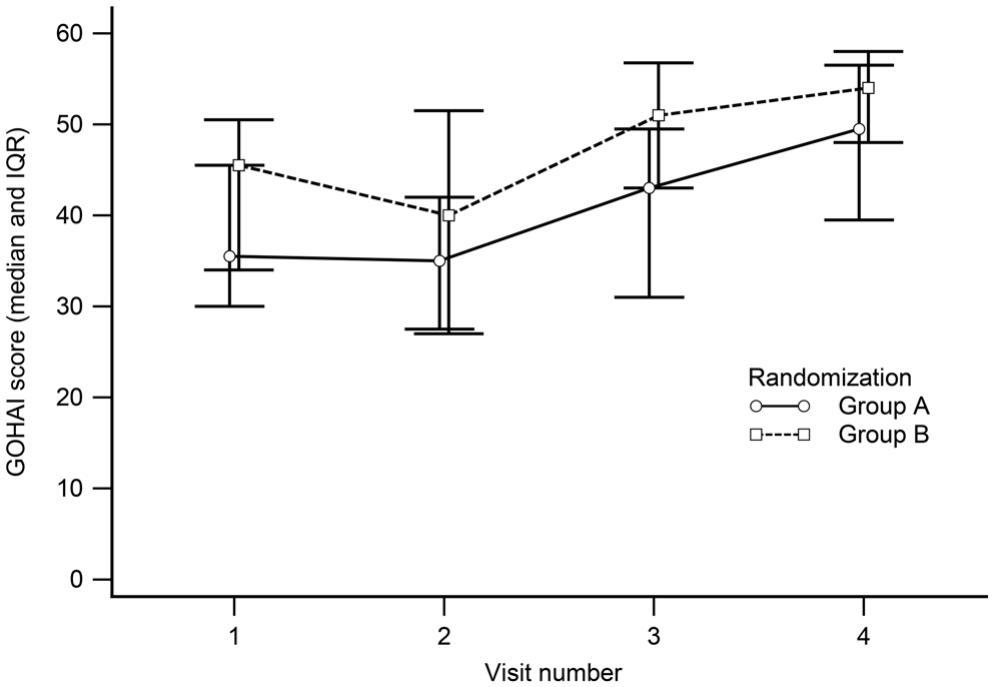
Changes in GOHAI scores across study visits and treatment Groups. Visit #1—baseline (pre‐treatment); Visit #2—implant placement surgery (immediate loading, Group B); Visit #3–6‐8‐week follow‐up (implant loading, Group A; control visit, Group B); and Visit #4–1‐year follow‐up. Group A—standard‐diameter implants; Group B—mini‐implants. Data are expressed as median and interquartile range (IQR).

**FIGURE 4:**
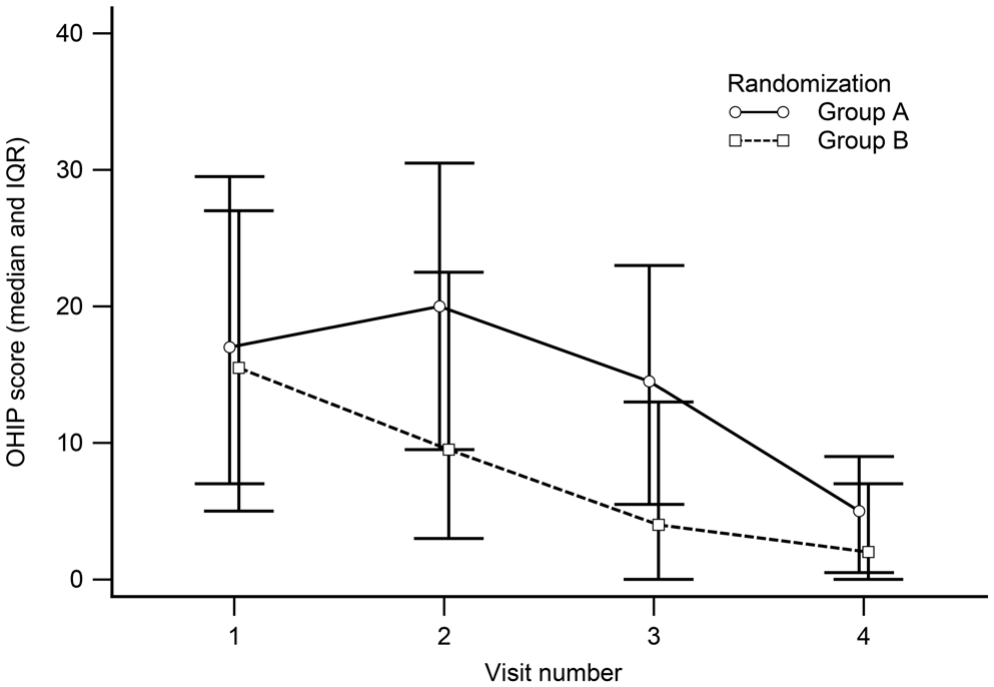
Changes in OHIP scores over time by study visit and treatment Group. Visit #1—baseline (pre‐treatment); Visit #2—implant placement surgery (immediate loading, Group B); Visit #3–6‐8‐week follow‐up (implant loading, Group A; control visit, Group B); and Visit #4–1‐year follow‐up. Group A—standard‐diameter implants; Group B—mini‐implants. Data are expressed as median and interquartile range (IQR).

**FIGURE 5:**
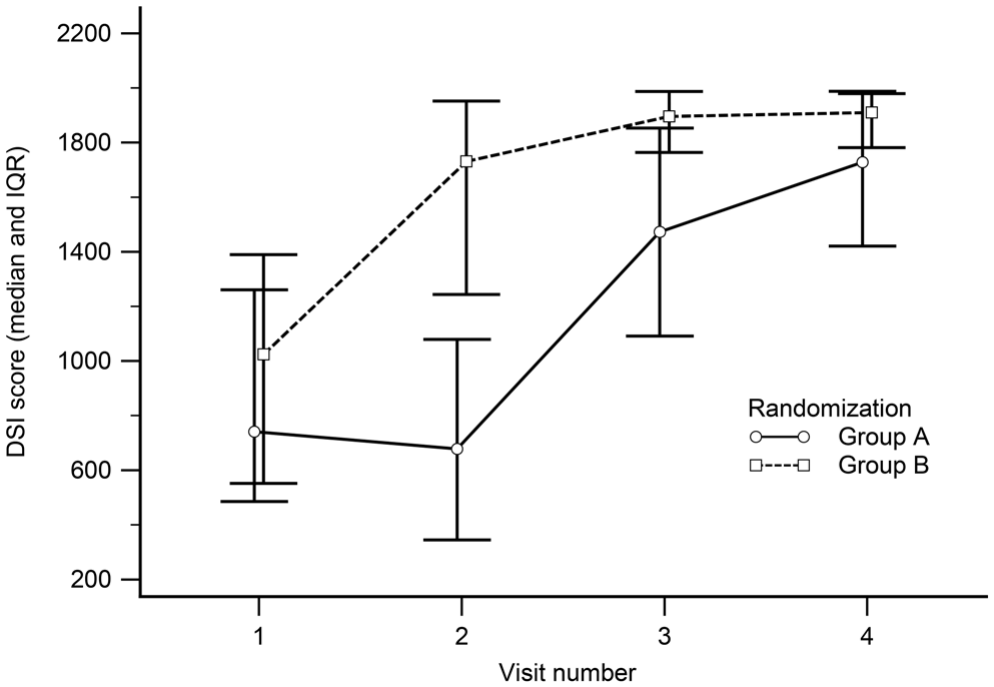
Changes in patient satisfaction across study visits and treatment Groups. Visit #1—baseline (pre‐treatment); Visit #2—implant placement surgery (immediate loading, Group B); Visit #3–6‐8‐week follow‐up (implant loading, Group A; control visit, Group B); and Visit #4–1‐year follow‐up. Group A—standard‐diameter implants; Group B—mini‐implants. Data are expressed as median and interquartile range (IQR).

Figures 3, 4, 5 present the summary data for the assessed outcomes—GOHAI, OHIP, and DSI. Since most of the data of time and treatment subgroups showed non‐normal distribution, the data were presented as median (interquartile range) and analyzed using nonparametric bivariate comparison tests.

Overall, both treatments showed a significant positive effect at the 1‐year follow‐up, compared with pretreatment values (*p* < 0.01), as assessed across the three outcome measures (Wilcoxon Signed Ranks Test). Moreover, Group B showed better outcomes at the intermediate time points after immediate loading and at the visit #3 follow‐up (Mann–Whitney test). Figures also suggest better outcome values for Group B at all intermediate post‐treatment assessments (*p* < 0.05). However, at the final 1‐year follow‐up, the two Groups appeared to perform similarly in terms of changes in GOHAI (*p* = 0.521), OHIP (*p* = 0.933), and DSI (*p* = 0.482) scores.

Moreover, analysis of the magnitude of the treatment effect (effect size) revealed a large effect size for all outcome parameters in Group A (0.62, 0.67, and 0.82) and Group B (0.46, 0.68, and 0.84) for GOHAI, OHIP, and DSI, respectively. Therefore, the highest positive effects were observed for patient satisfaction, followed by OHIP score, and a moderate to large effect for GOHAI.

The multiple regression analysis (Table 1) revealed that the two treatments had similar effects on the OHIP (*p* = 0.341) and DSI (*p* = 0.502) measures, while GOHAI was slightly better for Group B (*p* = 0.028). The effects of treatment were significant at the 1‐year follow‐up compared with baseline across the three measured outcomes (*p* < 0.001). However, when the interactions between treatment Groups and time points were assessed, the results suggest higher positive effects at the intermediary time points for Group B concerning patient satisfaction (*p* = 0.001), indicating a positive effect of the immediate loading of the mini‐implants. No effect of the study center was observed (*p* > 0.05), and this variable was not included in the final regression models.

**TABLE 1:** Multiple regression parameters for the treatment effects by study group, time point, and interaction.

Parameter	GOHAI		OHIP‐G14		DSI	
	B (SE)	*p*	B (SE)	*p*	B (SE)	*p*
Intercept	35.9 (2.41)	< 0.001	19.41 (2.50)	< 0.001	913.1 (96.8)	< 0.001
Treatment group						
Group B	6.72 (3.06)	0.028	−2.84 (3.27)	0.385	116.6 (137.2)	0.396
Group A	Reference		Reference		Reference	
Time points						
Visit 4	10.47 (2.76)	< 0.001	−10.75 (2.59)	< 0.001	736.6 (86.7)	< 0.001
Visit 3	2.44 (3.30)	0.460	−2.75 (2.28)	0.227	501.4 (108.5)	< 0.001
Visit 2	−4.06 (3.08)	0.187	2.31 (2.58)	0.370	−75.7 (97.9)	0.439
Visit 1	Reference		Reference		Reference	
Time points X Group (Reference: Group A, Visit 1)						
Group B Visit 4	−4.06 (4.40)	0.356	−0.78 (3.40)	0.819	79.4 (132.3)	0.548
Group B Visit 3	−1.22 (5.02)	0.808	−6.23 (3.28)	0.057	275.6 (148.3)	0.063
Group B Visit 2	−2.59 (4.44)	0.559	−4.72 (3.76)	0.209	546.8 (161.9)	0.001
Group B Visit 1	Reference		Reference		Reference	

## 4 Discussion

This study confirmed the hypothesis that mandibular IODs treatment with four one‐piece mini‐implants carrying stud‐type attachments and immediate loading provides quicker improved dPROs than standard treatment with two two‐piece implants and conventional loading in the first year after implant surgery. Immediate loading was associated with improvements in patients' condition shortly after implant placement, leading to a faster return to improved masticatory function and OHRQoL, and potentially reduced patient pain, thereby streamlining the overall rehabilitation process.

Other studies comparing protocols using mini‐ and conventional implants have reported that overdentures retained by 4 or 2 mini‐implants can achieve OHRQoL and satisfaction at least comparable to that of 2 conventional implants; however, with the caveat that mini‐implants have lower survival rates (De Souza et al. 2015). However, implant survival rates for the mini‐implants seem to be higher than older mini‐implant systems used in most of the previous studies, since a previous study using this novel Ti‐Zr mini‐implant showed 100% survival after 1 year, including cases with immediate loading protocol (Curado et al. 2023).

The advantages of the straightforward protocol for mini‐implants were reported in a previous study that tested the combined flapless surgery and immediate loading of mini‐implants (Leles et al. 2022) and noted that flapless surgery requires less clinical time and results in more straightforward intraoral prosthetic incorporation of attachments compared to flapped surgeries, while immediate loading did not increase the risk of early implant failure when satisfactory primary stability is achieved. Moreover, immediate loading resulted in lower post‐surgical complaint rates. In contrast, delayed conventional loading was associated with discontinuous use of the prosthesis and poor function during the implant healing period (Leles et al. 2022). The improved patient experience in the post‐surgical period may be responsible for the higher satisfaction and lower quality‐of‐life impacts observed 7–15 days after implant placement.

Regarding the primary stability parameter for immediate loading of this TiZr mini‐implant system, the manufacturer recommends a minimum final insertion torque of 35 Ncm for all implants. This study adopted the ≥ 25 Ncm cut‐off value to avoid deviations from the study protocol and to reduce attrition rates. This modification in the instructions for use, however, did not influence the risk of early implant failure in this study. Nevertheless, in routine clinical practice, the ≥ 35 Ncm limit for immediate loading should be strictly adhered to comply with the IFUs and, therefore, the manufacturer's guarantee. Conversely, conventional loading after at least 6 weeks of healing should be adopted when primary stability is not achieved for all implants.

A study with a similar design compared treatments with mandibular overdentures retained by four mini‐implants versus two standard‐size implants (Zygogiannis et al. 2018). Results showed that immediate loading was possible for all mini‐implant patients, but only for 60% of patients treated with standard‐diameter implants, and a significant improvement in the satisfaction and quality of life was observed after denture stabilization with implants, regardless of the type of implants and loading timing. Treatment Groups and loading time were similar at the 3‐ and 12‐month follow‐ups concerning treatment effects, corroborating that both treatments can offer patient improvement of equal magnitude (Zygogiannis et al. 2018).

In our study, after 1 year, both treatments seem to perform similarly, suggesting that they are suitable alternatives to improve the function of unstable mandibular dentures. However, since the study timeframe was limited to 1 year of overdenture use, more extended follow‐up periods may be needed to assess the effect of prosthetic complications and deterioration of oral function in older patients on the satisfaction with treatment, and to develop long‐term maintenance solutions if patients become frail because no easy downgrading approaches of one‐piece mini‐implants exist (Enkling et al. 2017).

The results from this study corroborate the use of mini‐implants for mandibular overdenture retention. Besides the advantages concerning patient perception in the short‐term, the use of mini‐implants may also be justified by the simplification of the intervention for patients demanding less invasive procedures, for whom straightforward procedures would be preferable, and for patients with limited bone width and who can often allow for immediate denture placement, leading to faster recovery and increased patient satisfaction (Park et al. 2023).

A potential limitation concerns the comparability of the PEEK retention inserts between the two attachment systems. Although the manufacturer documentation lists identical retention forces (≈300, 750, 1200, 1650, 2100 and 2550 g for the red, white, yellow, green, blue and black inserts, respectively) for both the Novaloc and the Optiloc system, the Optiloc patrix has a substantially smaller diameter (≈4.2 mm) than the Novaloc patrix (≈5.45 mm). It is therefore plausible that the absolute retention force per attachment is in fact lower for Optiloc than for Novaloc at any given colour code, and that the published values represent a transfer from the original Novaloc datasheet rather than independently measured Optiloc data. From a clinical perspective, however, this discrepancy is unlikely to have influenced our findings. Group B participants received four mini‐implants compared to two standard implants in Group A, so total prosthesis retention summed across attachments is unlikely to have been lower in the Optiloc group, even if the per‐attachment force were reduced. Furthermore, retention inserts were exchanged in response to patient‐reported insufficient retention rather than according to a predefined force threshold, and the distribution of insert colours at the one‐year follow‐up was comparable between groups. The colour code thus functioned as a relative step‐up in retention force within each system, and any absolute difference in Newtons between equivalent colours is unlikely to have meaningfully biased the patient‐reported OHRQoL outcomes (GOHAI, OHIP, DSI).

Regarding the OHRQoL instruments used in this study, both the OHIP and DSI questionnaires captured changes in patient perceptions following overdenture treatment and discriminated the potential advantages of the mini‐implant alternative in the short term. However, GOHAI only detected the positive effects of overdenture treatment at the one‐year follow‐up, and with a lower magnitude compared to the other instruments. This is consistent with other studies, which found that OHIP‐Edent showed higher discriminant validity than OHIP‐14 and GOHAI, as it was better able to identify patients with poor prosthetic status or unstable dentures (El Osta et al. 2021). Although both OHIP‐14 and GOHAI detect the impacts of oral disease in older adults and can be considered broadly equivalent, improvements in OHRQoL following treatment in older adults may be more challenging to detect with GOHAI (Gokturk and Yarkac 2019).

Concerning the limitations of this randomized clinical trial, participant‐related concerns may include limited generalizability of the findings, as the artificial environment of the trial and the specific characteristics of the study participants may not accurately reflect the diverse patient population that will use the treatment in “real world” settings. In addition, an underpowered study due to patient drop‐out may be considered, as missing data in longitudinal clinical studies can reduce statistical power and lead to biased estimates, resulting in invalid conclusions (Madley‐Dowd et al. 2019). However, the missing data after treatment provision occurred due to patient death (*n* = 3) or withdrawal (*n* = 1) and were within expected limits and may be considered negligible for the study.

There are other meaningful limitations of this randomized clinical trial. One is the inherent heterogeneity of treatment protocols and clinical settings between the two study groups. Although random allocation resulted in comparable baseline demographic characteristics, the interventions still differed in several important aspects beyond implant diameter and retentive parts. These differences included the number of implants (two versus four), the implant design (one‐piece versus two‐piece), the loading protocol (immediate versus conventional), and the overall clinical workflow, which may affect group comparability. In addition, despite the implementation of standardized methods and the identification of no influence of the study centers on the regression models, treatments were provided in two different types of clinical settings (university clinic and private practice), possibly causing uncontrolled contextual influences not addressed in this study.

Furthermore, group comparability may be compromised by differences in implants and attachment configurations, and these factors should be considered when interpreting results. In this study design, Group A received two implants with two Novaloc matrices; Group B received four implants with four miniaturized Optiloc matrices. The difference in the number of implants may have led to greater overall retention and stability in Group B and contributed to better short‐term patient‐reported outcomes after immediate loading of the implants.

Therefore, this study may have compared comprehensive treatment strategies rather than the effect of a single variable (e.g., an implant/retention system), thereby limiting causal attribution to any individual component of the study. Future clinical studies should include standardized attachment types and loading protocols, or use factorial designs to clarify the contributions of implant number, attachment retention, and loading protocol within the same study design (Leles et al. 2022).

Finally, the ultimate goal of this study was to compare the two treatment alternatives to evaluate which one is better recommended for edentulous patients. Findings confirmed that an implant mandibular overdenture, regardless of the type and number of implants used, is an effective approach for providing self‐perceived benefits to patients wearing a conventional mandibular denture. However, the decision on the type of implant and attachment system should consider individual aspects, such as biological conditions, costs, and patient preferences, among others (Leles et al. 2011). Future research focusing on economic analyses and subjective evaluations of the patient's experience could provide critical support for more accurate decision‐making in clinical practice.

## 5 Conclusion

Within the limitations of this randomized clinical two‐center trial, significant improvements in patient satisfaction and oral health‐related quality of life were observed after 1 year following conversion from conventional dentures to overdentures. The results also suggest that the four mini‐implant group reported slightly better outcomes compared to the two‐implant group, especially at shorter‐term follow‐ups due to the immediate loading of the implants. The findings from this study support the protocol using four Ti mini‐implants as a suitable treatment alternative for the edentulous mandible.

## Author Contributions

**Clemens Raabe:** methodology, investigation, visualization, writing – original draft, writing – review and editing, data curation. **Beat Wallkamm:** methodology, investigation, validation, writing – original draft, supervision. **Cláudio Rodrigues Leles:** formal analysis, writing – original draft, writing – review and editing, validation. **Valérie G. A. Suter:** investigation, validation, supervision, visualization, writing – original draft, data curation. **Martin Schimmel:** conceptualization, investigation, funding acquisition, writing – original draft, writing – review and editing, resources, project administration, methodology. **Nicole Schenk:** investigation, validation, writing – original draft, data curation. **Simone F. M. Janner:** methodology, investigation, validation, writing – original draft. **Samir Abou‐Ayash:** conceptualization, methodology, investigation, resources, writing – original draft, writing – review and editing.

## Funding

This work was supported by the International Team for Implantology, ITI Grant 1286_2018.

## Conflicts of Interest

Martin Schimmel, Cláudio R. Leles, Samir Abou‐Ayash, Clemens Raabe, and Simone F M Janner are the recipients of various grants as main and co‐applicants from Institut Straumann AG and the ITI. They occasionally lecture for Institut Straumann AG, Switzerland. The other authors do not report any conflicts of interest related to the present study.

## Supporting information


**Data S1:** clr70143‐sup‐0001‐DataS1.pdf.

## Data Availability

The data that support the findings of this study are available from the corresponding author upon reasonable request.
